# Weekend effect in emergency hospitalizations of older adults in Hong Kong

**DOI:** 10.1038/s41598-026-54977-0

**Published:** 2026-05-26

**Authors:** Hong-jie Yu, Eric Tsz-Chun Lai, Kuen Lam, Irene Yuk-Ying Ho, Grace Lai-Hung Wong, Jean Woo

**Affiliations:** 1 Institute of Health Equity, the Chinese University of Hong Kong, Hong Kong, Hong Kong SAR; 2 Jockey Club Institute of Ageing, the Chinese University of Hong Kong, Hong Kong, Hong Kong SAR; 3 Department of Medicine & Therapeutics, Faculty of Medicine, the Chinese University of Hong Kong, Hong Kong, Hong Kong SAR; 4 Department of Medicine and Geriatrics, Shatin Hospital, Hong Kong, Hong Kong SAR; 5 Medical Data Analytics Centre, The Chinese University of Hong Kong, Hong Kong, Hong Kong SAR

**Keywords:** Weekend effect, Emergency departments, Hospital mortality, Hospital readmission, Diseases, Health care, Medical research, Risk factors

## Abstract

The impact of the weekend effect on hospitalization outcomes remains debated. This study evaluated the “weekend effect” by defining two distinct patterns based on admission–discharge timing: “weekend-admission/discharge” (either admission or discharge occurring on weekends) and “weekday-admission-discharge” (both admission and discharge occurring on weekdays). Utilizing 3,447,877 emergency hospitalization spells among older patients in Hong Kong from 2012 to 2021, multivariable logistic regression with a random effect of hospital revealed that the weekend-admission/discharge pattern accounted for 42.5% of hospitalizations and was associated with significantly higher in-hospital mortality (5.0% vs. 3.8%; adjusted odds ratio [aOR] = 1.359, 95% CI: 1.344–1.373). Paradoxically, this pattern demonstrated minimally protective effects for post-discharge outcomes, with lower 30-day mortality (aOR = 0.963, 95% CI: 0.948–0.978) and reduced 30-day emergency readmission risk (aOR = 0.986, 95% CI: 0.979–0.992). Subgroup analyses confirmed consistent elevation of in-hospital mortality risk across all spell groups, while revealing substantial variations by length of stay, clinical specialty, and principal diagnosis. The protective post-discharge effects were predominantly observed among those with longer hospitalizations. These findings demonstrate a complex duality in weekend care outcomes and suggest that the weekend effect reflects systemic care integration challenges and selective discharge patterns rather than simple temporal variations in care quality. Future quality improvement initiatives should focus on enhancing care continuity and developing targeted approaches for high-risk clinical contexts. Meanwhile, more research is needed to elucidate the underlying mechanisms driving these paradoxical outcomes.

**Supplementary Information:**

The online version contains supplementary material available at 10.1038/s41598-026-54977-0.

## Introduction

Previous studies showed that the “weekend effect” related to hospital admissions was associated with an elevated risk of mortality^1–8^. Moreover, recent studies have expanded this concept to encompass hospital discharges, suggesting that a similar heightened mortality risk may also be present in this context^9,10^. This growing body of evidence not only reinforces the significance of the weekend effect of admissions and discharges on mortality risk, but also broadens its implications to include various clinical outcomes, such as prolonged length of stay (LOS), increased readmission rates, and a higher incidence of complications^6,11–13^. Existing literature on the weekend effect has predominantly focused on Western healthcare contexts, particularly in the UK and the US^5–7,13,14^. However, no studies to date have assessed the weekend effect within the unique healthcare context of Hong Kong, where the healthcare system is substantially challenged by a rapidly aging population^14^.

The primary hypothesis guiding the investigation of the weekend effect is based on the disparities in medical services available during weekdays compared with weekends, including reduced staffing levels, lower rates of invasive procedures, and limited access to diagnostic tests and treatment options on weekends^7,13,15^. Within this hypothesis, weekends are expected to influence all hospitalizations, including those admitted on weekdays with extended weekend stays, rather than only impacting new admissions or those nearing discharge on weekends^16,17^. A large population-based cohort study involving 323,895 hospitalizations in Canada revealed that weekend stays also faced an increased risk of mortality, a risk that persists even independent of the weekend admission^17^. This finding suggests that both the timing of admissions and discharges on weekends, as well as the duration of hospital stays that span the weekend, may be related to adverse clinical outcomes, highlighting the need for an integrated understanding of admission, stay, and discharge to clarify the weekend effect.

Prior studies examining the weekend effect typically compare clinical outcomes between weekend and weekday admissions or discharges, focusing on isolated time points such as the day of admission or the day of discharge^5,6,13,18^. However, such a fragmented approach fails to capture the inherent weekly cyclicality embedded in the entire admission–discharge process. In reality, hospital stays follow systematic modulo-7 patterns determined by LOS and days of admission and discharge, which inherently shape whether a patient is admitted or discharged on a weekend. For example, a 1-day LOS can result in weekend exposure, with admissions on Friday, Saturday, or Sunday leading to discharges on Saturday, Sunday, or Monday, respectively. Similarly, an 8-day LOS results in same-day-of-week admission-discharge patterns: Friday, Saturday, Sunday admissions lead to Saturday, Sunday, Monday discharges the following week, respectively. These patterns recur cyclically across LOS strata (e.g., modulo-7 residues), creating varying probabilities of weekend exposure not fully accounted for in conventional weekday/weekend dichotomies. Therefore, to more accurately assess the weekend effect across the care continuum, we define hospitalization by whether they involve weekend admission or discharge, thereby incorporating the structured temporal interplay between admission day, LOS, and discharge day inherent in the weekly cycle. By comparing all-cause in-hospital mortality and post-discharge emergency readmission and mortality risks between weekday admission and discharge and weekend admission or discharge hospitalization patterns (herein after referred as weekday-admission-discharge vs. weekend admission/discharge), we aimed to provide a clearer and more comprehensive understanding of the weekend effect on hospitalization outcomes.

## Methods

### Study design and data source

This retrospective analysis utilized data obtained from the Clinical Data Analysis and Reporting System (CDARS), which is managed by the Hospital Authority (HA) of Hong Kong. The HA is a statutory body responsible for overseeing 43 public hospitals (18 currently offer Accident and Emergency care services), 49 specialist clinics, and 73 general outpatient clinics throughout the region^19^. The CDARS serves as a comprehensive electronic database that consolidates extensive patient information, encompassing demographic details, mortality records, diagnoses, procedures, medication prescriptions, and laboratory results from all public healthcare institutions in Hong Kong. This database represents approximately 80% of hospital admissions in the territory. The integrity of the data within CDARS has been rigorously validated and has been utilized in a variety of epidemiological research studies^19,20^.

### Study population

All older adults (aged ≥ 65 years) who admitted to the emergency departments of public hospitals in Hong Kong from 2012 to 2021 were included. From 3,659,800 episodes, we excluded 83,120 day patients who were admitted and discharged on the same day (Supplementary Fig. 1). This exclusion was necessary to specifically investigate the weekend effect within the inpatient setting, as the outcomes for day-case patients are primarily influenced by pre-hospital factors unrelated to inpatient care quality^21^. To measure continuous hospital stays for patients who transferred between consultants or hospitals^22^, we consolidated inter- and intra-hospital transfers during continuous stays into a single spell, yielding 3,554,777 spells. Hospitalization spells were classified into two patterns based on the timing of admission and discharge: “weekday admission with weekday discharge (weekday-admission-discharge)” pattern and “weekend admission or discharge (weekend-admission/discharge)” pattern. This binary classification intentionally aggregates any hospitalization involving a weekend care transition. As detailed in Supplementary Table 1, the probability of weekend exposure follows a predictable modulo-7 pattern determined by admission day and LOS. This approach creates comparison groups systematically balanced across LOS strata, circumventing the structural confounding that would arise from analyzing admission and discharge timing as independent categories. Moreover, LOS serves as a proxy for patient-related factors and significantly influences hospitalization outcomes, with longer LOS being associated with increased risks of mortality and readmission^23–25^. To mitigate the potential skewing effects caused by complex conditions or complications unrelated to the initial admission but associated with prolonged LOS^26^, we excluded spells with an LOS exceeding 28 days, resulting in an attrition rate of 3.0%.

### Measurement

#### Outcome

The primary outcome of this study was all-cause in-hospital mortality, operationalized as a patient having a registered date of death that fell within the interval from admission to discharge, inclusive. Secondary outcomes encompassed all-cause emergency readmission and mortality within 7 and 30 days following discharge. These timeframes were selected in alignment with established literature to facilitate comparative interpretation of our findings^13,27,28^. All post-discharge outcomes were delineated with reference to the index admission, which was identified as either the first recorded hospitalization or an admission occurring more than 30 days after a previous discharge^29^. In the event of multiple readmissions within the specified window, only the first was considered. Furthermore, to account for the competing risk of death, individuals who experienced post-discharge mortality within either the 7- or 30-day period were excluded from the analysis of readmission at the corresponding time point.

#### Covariates

The covariates included LOS, age, sex, living arrangements, payment source, season of admission, principal diagnosis, and clinical specialty. LOS, defined as the interval between the discharge date and the admission date, was classified into four strata based on a repeating 7-day pattern. Sex (male/female), old-age home residence (yes/no), and payment source (public assistance/non-public assistance, indicating financial difficulty) were treated as binary variables, while seasons were categorized from Spring to Winter. Clinical specialty indicated the triage of emergency patients to various specialties, with the top four identified as Emergency Medicine, Internal Medicine, Orthopedics, and Surgery; all other specialties were aggregated into an “Other” category. Principal diagnoses were coded according to the 10th revision of the International Classification of Diseases (ICD-10), with any diagnoses accounting for less than 2% of total cases also categorized as “Other” category.

### Statistical analysis

Baseline characteristics were compared using Wilcoxon tests for continuous variables and Chi-square tests for categorical variables. We employed multivariable logistic regression with hospital-level random effects to assess associations between hospitalization patterns (weekday-admission-discharge versus weekend-admission/discharge) and outcomes. Model 1 adjusted LOS considering weekend admission or discharge is structurally influenced by LOS, while Model 2 additionally adjusted for age, sex, old-age home residence, season, payment source, clinical specialty, and principal diagnosis. Readmission analyses excluded patients who died during hospitalization or within the specified post-discharge period. Subgroup analyses based on those covariates were also performed to explore potential moderators between association between weekend effect and hospitalization outcomes. For example, residents of old-age homes experience higher mortality rates and increased disease complexity, and they are particularly frailer than their community-dwelling counterparts^30^, hypothesizing that weekend effect would be particularly pronounced among these residents. Furthermore, considering that mortality rates among older adults are typically lowest in the summer and highest in the winter^31^, we proposed that the disparity in mortality risk between the two hospitalization patterns might be exacerbated during the winter months due to seasonal variations. Moreover, subgroup analyses of LOS strata, clinical specialty, and principal diagnosis were conducted to potentially address how LOS duration and hospital care system difference affect the weekend effect on hospitalization outcomes.

To enhance the robustness of our findings, we conducted three additional sensitivity analyses. First, we performed propensity score matching using 1:1 nearest-neighbor matching with a caliper of 0.2 to address the imbalance in covariates between the two hospitalization patterns. Second, we excluded holidays to account for the potential effect of holiday admissions on readmission and mortality risk^28^. Third, recognizing that a single patient may have multiple spells over a follow-up period of up to ten years, we randomly selected only one spell per patient for analysis. All analyses were performed using *R* software (Version 4.4.1), and a two-sided *p*-value of less than 0.05 was deemed statistically significant.

### Ethics approval and consent to participate

The study was conducted in accordance with the Declaration of Helsinki. This research, along with the waiver of individual patient informed consent, received ethical approval from the Joint Chinese University of Hong Kong-New Territories East Cluster Clinical Research Ethics Committee (Reference Number: 2022.157; Approval Date: June 2, 2022).

## Results

The study included 3,447,877 eligible emergency hospitalizations for adults aged 65 years and older. The cohort had a median age of 81 years, with 51% female patients, 24% residing in old-age homes, and 43% receiving public assistance. The median LOS was 4 days, with 81% of hospitalizations lasting less than one week. Internal Medicine accounted for most admissions (63%), and the most frequent principal diagnoses were symptoms and illness-defined diseases (R00-R99; 22%), respiratory diseases (J00-J99; 18%), and circulatory diseases (I00-I99; 14%). Notably, Sunday accounted for only 6.9% of all discharges (*n* = 237,830) despite a relatively uniform distribution of admissions and in-hospital deaths by day of the week (Supplementary Table 2), and in-hospital mortality increased progressively beyond a LOS of two days. Among these hospitalizations, 1,462,767 (42.5%) followed a weekend-admission/discharge pattern (Table 1). When analysis was restricted to index hospitalizations to minimize potential bias from correlated observations within patients who had multiple hospitalizations, the cohort included 2,682,852 admissions, with 1,132,408 (42.2%) in the weekend-admission/discharge group (Table 2).

**Table 1:** Characteristics of eligible emergency hospitalizations by admission and discharge day of the week.

Characteristics of eligible hospitalizations*	Weekday admission and discharge (*N* = 1985110)	Weekend admission or discharge (*N* = 1462767)	*p*-value^†^
Weekend admission			< 0.001
No	1,985,110 (100%)	525,125 (35.9%)	
Yes	0 (0%)	937,642 (64.1%)	
Weekend discharge			< 0.001
No	1,985,110 (100%)	815,044 (55.7%)	
Yes	0 (0%)	647,723 (44.3%)	
In-hospital death			< 0.001
No	1,909,211 (96.2%)	1,389,572 (95.0%)	
Yes	75,899 (3.8%)	73,195 (5.0%)	
Age, *years*	81 (74–87)	81 (74–87)	< 0.001
Youngest (65–74 *years*)	545,554 (27.5%)	391,073 (26.7%)	< 0.001
Middle (75–84 *years*)	741,780 (37.4%)	547,055 (37.4%)	
Oldest (≥ 85 *years*)	697,776 (35.2%)	524,639 (35.9%)	
Sex			0.079
Female	1,015,619 (51.2%)	749,781 (51.3%)	
Male	969,491 (48.8%)	712,986 (48.7%)	
Old-age home residence			< 0.001
No	1,526,056 (76.9%)	1,104,027 (75.5%)	
Yes	459,054 (23.1%)	358,740 (24.5%)	
Publicly-assisted payment			< 0.001
No	1,141,963 (57.5%)	837,431 (57.2%)	
Yes	843,147 (42.5%)	625,336 (42.8%)	
Admission year			< 0.001
2012	179,566 (9.0%)	135,495 (9.3%)	
2013	179,639 (9.0%)	134,474 (9.2%)	
2014	185,565 (9.3%)	139,136 (9.5%)	
2015	193,291 (9.7%)	143,778 (9.8%)	
2016	198,650 (10.0%)	150,895 (10.3%)	
2017	212,572 (10.7%)	156,621 (10.7%)	
2018	217,099 (10.9%)	158,008 (10.8%)	
2019	223,164 (11.2%)	163,801 (11.2%)	
2020	185,172 (9.3%)	131,228 (9.0%)	
2021	210,392 (10.6%)	149,331 (10.2%)	
Admission season			< 0.001
Spring	503,455 (25.4%)	374,044 (25.6%)	
Summer	484,791 (24.4%)	353,991 (24.2%)	
Fall	480,888 (24.2%)	350,054 (23.9%)	
Winter	515,976 (26.0%)	384,678 (26.3%)	
Length of stay, *days*	4 (2–7)	3 (2–5)	< 0.001
Stratum 1 (1–7 days)	1,587,400 (80.0%)	1,184,174 (81.0%)	< 0.001
Stratum 2 (8–14 days)	271,561 (13.7%)	188,625 (12.9%)	
Stratum 3 (15–21 days)	86,528 (4.4%)	61,698 (4.2%)	
Stratum 4 (22–28 days)	39,621 (2.0%)	28,270 (1.9%)	
Clinical specialty			< 0.001
Emergency Medicine	218,320 (11.0%)	175,055 (12.0%)	
Internal Medicine	1,240,568 (62.5%)	922,020 (63.0%)	
Orthopedics	187,009 (9.4%)	111,824 (7.6%)	
Surgery	288,351 (14.5%)	217,414 (14.9%)	
Others	50,862 (2.6%)	36,454 (2.5%)	
Principal diagnosis^#^			< 0.001
A00-B99: Certain infectious and parasitic diseases	77,148 (3.9%)	60,716 (4.2%)	
C00-D48: Neoplasms	71,619 (3.6%)	51,724 (3.5%)	
E00-E90: Endocrine, nutritional and metabolic diseases	88,729 (4.5%)	64,353 (4.4%)	
I00-I99: Diseases of the circulatory system	279,781 (14.1%)	198,497 (13.6%)	
J00-J99: Diseases of the respiratory system	340,309 (17.1%)	266,211 (18.2%)	
K00-K93: Diseases of the digestive system	164,598 (8.3%)	119,701 (8.2%)	
M00-M99: Diseases of the musculoskeletal system and connective tissue	107,720 (5.4%)	72,491 (5.0%)	
N00-N99: Diseases of the genitourinary system	111,643 (5.6%)	84,665 (5.8%)	
R00-R99: Symptoms, signs and abnormal clinical and laboratory findings, not elsewhere classified	421,080 (21.2%)	323,365 (22.1%)	
S00-T98: Injury, poisoning and certain other consequences of external causes	160,230 (8.1%)	112,127 (7.7%)	
Others	162,253 (8.2%)	108,917 (7.4%)	

*Eligible hospitalizations were defined as emergency admissions from older adults (aged ≥ 65 years) who had at least one overnight stay in the hospital. We further excluded prolonged hospitalization spells with a length of stay of ≥ 28 days.

^†^The differences between the two hospitalization patterns were assessed using the Wilcoxon test for age and length of stay, and the Chi-square test for all categorical variables.

^#^Principal diagnosis was based on the 10th revision of the International Classification of Diseases (ICD-10).

**Table 2:** Characteristics of index emergency hospitalizations by admission and discharge day of the week.

Characteristics of index hospitalizations*	Weekday admission and discharge (*N* = 1550444)	Weekend admission or discharge (*N* = 1132408)	*p*-value^†^
Weekend admission			< 0.001
No	1,550,444 (100%)	408,336 (36.1%)	
Yes	0 (0%)	724,072 (63.9%)	
Weekend discharge			< 0.001
No	1,550,444 (100%)	628,836 (55.5%)	
Yes	0 (0%)	503,572 (44.5%)	
Mortality at 30-day post-discharge			0.048
No	1,541,792 (99.4%)	1,126,294 (99.5%)	
Yes	8652 (0.6%)	6114 (0.5%)	
Mortality at 7-day post-discharge			< 0.001
No	1,509,168 (97.3%)	1,103,171 (97.4%)	
Yes	41,276 (2.7%)	29,237 (2.6%)	
Emergency readmission at 30-day post-discharge			0.612
No	1,454,817 (93.8%)	1,062,736 (93.8%)	
Yes	95,627 (6.2%)	69,672 (6.2%)	
Emergency readmission at 7-day post-discharge			0.028
No	1,274,402 (82.2%)	931,973 (82.3%)	
Yes	276,042 (17.8%)	200,435 (17.7%)	
Age, *years*	81 (73–87)	81 (73–87)	< 0.001
Youngest (65–74 *years*)	449,938 (29.0%)	320,416 (28.3%)	< 0.001
Middle (75–84 *years*)	581,274 (37.5%)	425,013 (37.5%)	
Oldest (≥ 85 *years*)	519,232 (33.5%)	386,979 (34.2%)	
Sex			0.013
Female	801,207 (51.7%)	586,914 (51.8%)	
Male	749,237 (48.3%)	545,494 (48.2%)	
Old-age home residence			< 0.001
No	1,239,898 (80.0%)	891,584 (78.7%)	
Yes	310,546 (20.0%)	240,824 (21.3%)	
Publicly-assisted payment			0.01
No	927,683 (59.8%)	675,778 (59.7%)	
Yes	622,761 (40.2%)	456,630 (40.3%)	
Admission year			< 0.001
2012	141,803 (9.1%)	106,583 (9.4%)	
2013	139,950 (9.0%)	103,789 (9.2%)	
2014	144,695 (9.3%)	107,349 (9.5%)	
2015	150,386 (9.7%)	111,052 (9.8%)	
2016	154,181 (9.9%)	116,091 (10.3%)	
2017	165,728 (10.7%)	121,175 (10.7%)	
2018	169,325 (10.9%)	122,072 (10.8%)	
2019	174,337 (11.2%)	126,779 (11.2%)	
2020	145,117 (9.4%)	101,574 (9.0%)	
2021	164,922 (10.6%)	115,944 (10.2%)	
Admission season			< 0.001
Spring	390,394 (25.2%)	287,038 (25.3%)	
Summer	376,725 (24.3%)	272,400 (24.1%)	
Fall	377,367 (24.3%)	272,078 (24.0%)	
Winter	405,958 (26.2%)	300,892 (26.6%)	
Length of stay, *days*	3 (2–7)	3 (2–5)	< 0.001
Stratum 1 (1–7 days)	1,253,875 (80.9%)	927,112 (81.9%)	< 0.001
Stratum 2 (8–14 days)	203,158 (13.1%)	139,065 (12.3%)	
Stratum 3 (15–21 days)	64,081 (4.1%)	45,358 (4.0%)	
Stratum 4 (22–28 days)	29,330 (1.9%)	20,873 (1.8%)	
Clinical specialty			< 0.001
Emergency Medicine	186,443 (12.0%)	148,594 (13.1%)	
Internal Medicine	936,518 (60.4%)	689,424 (60.9%)	
Orthopedics	163,369 (10.5%)	98,505 (8.7%)	
Surgery	224,046 (14.5%)	167,167 (14.8%)	
Others	40,068 (2.6%)	28,718 (2.5%)	
Principal diagnosis^#^			< 0.001
A00-B99: Certain infectious and parasitic diseases	59,713 (3.9%)	47,073 (4.2%)	
C00-D48: Neoplasms	47,613 (3.1%)	33,537 (3.0%)	
E00-E90: Endocrine, nutritional and metabolic diseases	67,244 (4.3%)	48,256 (4.3%)	
I00-I99: Diseases of the circulatory system	221,457 (14.3%)	155,683 (13.7%)	
J00-J99: Diseases of the respiratory system	249,387 (16.1%)	193,819 (17.1%)	
K00-K93: Diseases of the digestive system	130,524 (8.4%)	93,991 (8.3%)	
M00-M99: Diseases of the musculoskeletal system and connective tissue	91,004 (5.9%)	61,099 (5.4%)	
N00-N99: Diseases of the genitourinary system	80,851 (5.2%)	60,897 (5.4%)	
R00-R99: Symptoms, signs and abnormal clinical and laboratory findings, not elsewhere classified	328,618 (21.2%)	250,484 (22.1%)	
S00-T98: Injury, poisoning and certain other consequences of external causes	141,881 (9.2%)	98,999 (8.7%)	
Others	132,152 (8.5%)	88,570 (7.8%)	

*Index hospitalizations were defined as either the first hospitalization or those with a prior discharge occurring more than 30 days before admission.

^†^The differences between the two hospitalization patterns were assessed using the Wilcoxon test for age and length of stay, and the Chi-square test for all categorical variables.

^#^Principal diagnosis was based on the 10th revision of the International Classification of Diseases (ICD-10).

Comparative analysis revealed substantial differences between these two hospitalization patterns. Patients with weekend-admission/discharge had significantly shorter LOS (median 3 days, IQR 2–5) compared with those with weekday-admission-discharge (median 4 days, IQR 2–7). Clinical specialty distribution also differed notably, with the weekend-admission/discharge group having a lower proportion of orthopedic cases (7.6% vs. 9.4%) but a higher proportion of emergency medicine cases (12.0% vs. 11.0%). The weekend-admission/discharge group experienced higher in-hospital mortality (5.0% vs. 3.8%, *p*-value < 0.001, Table 1), yet demonstrated slightly lower post-discharge mortality (Table 2) at both 7 days (2.6% vs. 2.7%, *p*-value < 0.001) and 30 days (0.5% vs. 0.6%, *p*-value = 0.048). All-cause emergency readmission rates showed minimal differences, with no significant variation in 30-day readmission (6.2% for both groups) though a small difference was observed for 7-day readmission (17.7% vs. 17.8%, *p* = 0.028).

After multivariable adjustment, this complex association persisted (Table 3). The weekend-admission/discharge pattern was associated with 35.9% higher odds of in-hospital mortality (adjusted Odds Ratio [aOR] = 1.359, 95% CI: 1.344–1.373). In contrast, the association reversed following discharge. While adjustment for LOS alone yielded null associations, the fully adjusted model revealed that the weekend-admission/discharge pattern was associated with significantly lower odds of 7-day (aOR = 0.960, 95% CI: 0.928–0.992) and 30-day (aOR = 0.963, 95% CI: 0.948–0.978) post-discharge mortality. For all-cause emergency readmission, the weekend-admission/discharge pattern was associated with a small but significant reduction in the odds of 30-day readmission (aOR = 0.986, 95% CI: 0.979–0.992), but not 7-day readmission.

**Table 3:** Association between weekend admissions or discharges and the risk of mortality and emergency readmission.

Outcomes	Model 1^*^		Model 2^*^	
	Odds ratio (95% Confidence interval)	*p*-value	Odds ratio (95% Confidence interval)	*p*-value
In-hospital mortality	1.381 (1.366–1.395)	< 0.001	1.359 (1.344–1.373)	< 0.001
Morality at 7-day post-discharge	0.993 (0.961–1.027)	0.693	0.960 (0.928–0.992)	0.015
Morality at 30-day post-discharge	0.994 (0.979–1.009)	0.409	0.963 (0.948–0.978)	< 0.001
Emergency readmission at 7-day post-discharge^†^	1.004 (0.994–1.014)	0.463	0.993 (0.983–1.003)	0.160
Emergency readmission at 30-day post-discharge^†^	0.999 (0.993–1.006)	0.772	0.986 (0.979–0.992)	< 0.001

*The association between weekend admissions or discharges and the risk of mortality and readmission was evaluated using a logistic regression model that included a random effect for hospitals. Model 1 adjusted for length of stay, while Model 2 additionally adjusted for age, sex, old-age home residence, payment source, admission season, clinical specialty, and principal diagnosis.

^†^Readmission risks were assessed after excluding deaths occurring within 7 or 30 days to eliminate competing risks.

**Fig. 1:**
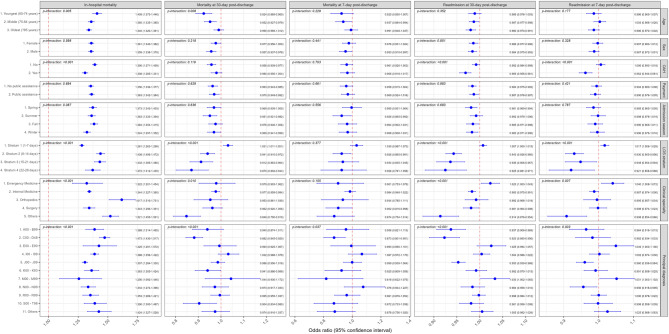
Subgroup analysis for the association between weekend admissions or discharges and the risk of mortality and emergency readmission. **Note**: The association between weekend admissions or discharges and the risk of mortality and emergency readmission was evaluated using a logistic regression model that included a random effect for hospitals and adjusted for length of stay age, sex, old-age home residence, payment source, admission season, clinical specialty, and principal diagnosis as appropriate. Emergency readmission risks were assessed after excluding deaths occurring within 7 or 30 days to eliminate competing risks. The *p*-values for interaction terms were calculated using likelihood ratio tests comparing models with and without each interaction term, while maintaining all other covariates in the model. OAH: Old-age homes; A00-B99: Certain infectious and parasitic diseases; C00-D48: Neoplasms; E00-E90: Endocrine, nutritional and metabolic diseases; I00-I99: Diseases of the circulatory system; J00-J99: Diseases of the respiratory system; K00-K93: Diseases of the digestive system; M00-M99: Diseases of the musculoskeletal system and connective tissue; N00-N99: Diseases of the genitourinary system; R00-R99: Symptoms, signs and abnormal clinical and laboratory findings, not elsewhere classified; S00-T98: Injury, poisoning and certain other consequences of external causes.

Subgroup analyses (Fig. 1) demonstrated that while the elevated in-hospital mortality risk associated with weekend-admission/discharge was consistent across all subgroups, the effect size was modified by age, old-age home residence, LOS stratum, clinical specialty, and principal diagnosis (*p*-interaction < 0.05). The effect was less pronounced in older adults, old-age home residents, and patients with short LOS (1–7 days), but most substantial in Orthopedics (aOR 1.617, 95% CI 1.510–1.731) and among patients with neoplasms (aOR 1.473, 95% CI 1.430–1.517). Post-discharge outcomes revealed striking variation by LOS: hospitalizations lasting 1–7 days showed neutral or increased risks, while longer stays (8–14, 15–21, and 22–28 days) demonstrated consistent protective effects that intensified with increasing hospitalization duration. Effects were also modified by clinical specialty and principal diagnosis, though most estimates were close to null despite statistical significance.

Multiple sensitivity analyses confirmed the robustness of these findings (Supplementary Table 3). Across propensity score matching, exclusion of public holidays, and random selection of single hospitalizations per patient, the weekend-admission/discharge pattern consistently demonstrated increased in-hospital mortality alongside decreased 30-day post-discharge mortality and emergency readmission. These analyses further revealed that protective post-discharge effects were primarily driven by hospitalizations exceeding one week and varied substantially across clinical specialties, being most pronounced in Surgery while absent or reversed in Emergency Medicine (Supplementary Table 4).

## Discussion

This territory-wide study utilizing ten years of Hong Kong emergency department data presents a nuanced understanding of the “weekend effect” in older adult hospitalizations. By comparing two distinct care pathways, weekday-admission-discharge versus weekend-admission/discharge, we identified a paradoxical pattern: while exposure to weekend care transitions significantly increased the risk of in-hospital mortality, it was associated with minimally improved post-discharge outcomes including both reduced all-cause mortality and emergency readmission rates at 30 days.

The elevated in-hospital mortality risk (35.9% higher odds) associated with weekend-admission/discharge aligns with substantial existing literature documenting the weekend effect for either admission or discharge alone^2,3,9^. The increased in-hospital mortality risk likely stems from well-documented systemic limitations during weekends, including reduced staffing levels, limited availability of specialized diagnostics, and constrained access to invasive procedures^5,7,13,18^. These constraints also defer stable discharges to weekdays; consequently, weekend discharge volumes are substantially lower while in-hospital deaths remain evenly distributed, producing a denominator compression that mechanically inflates in-hospital mortality rates on weekend. The clinical manifestation of these systemic challenges is further reflected in a higher proportion of non-specific diagnoses (R00-R99) within the weekend-admission/discharge group, underscoring that current diagnostic framework, primarily organized around specific organ systems, may be particularly inadequate for capturing the complex, multi-system “geriatric syndromes” that commonly precipitate emergency hospitalization in older adults^32^.

The most intriguing finding, the protective effect for post-discharge mortality and emergency readmission, appears to be driven primarily by patient selection mechanisms, specifically the “depletion of susceptible” phenomenon^33^. In this paradigm, the well-documented suboptimal weekend care environment results in earlier mortality among the most critically ill and vulnerable patients during their hospitalization. Consequently, the cohort that survives to discharge following a weekend-admission/discharge represents a selectively resilient “survivor” population with inherently lower baseline risk for post-discharge adverse events. In contrast, the weekday-admission-discharge pattern likely includes a greater proportion of medically complex, frail patients whose conditions were stabilized during a longer hospitalization but who remain at elevated risk for post-discharge deterioration. Our stratified analysis by LOS strongly supports this. The elevated mortality and emergency readmission risks associated with the weekend effect are concentrated in the shortest LOS stratum (1–7 days), precisely where early deaths from the most acute illnesses would occur. In contrast, the protective effect emerges and strengthens in longer LOS strata (≥ 8 days). Another potential explanation is that clinicians may selectively prioritize weekend discharges for patients perceived as low-risk, mitigating emergency readmission probability^12,34^. Furthermore, the temporal proximity of weekend discharge may enhance familial or caregiver availability, facilitating smoother care transitions, a factor not consistently captured in administrative datasets^12^.

The relationship between weekend care transitions and patient outcomes demonstrates remarkable complexity when examined through the lens of patient subgroups. Our analysis further reveals that factors such as clinical specialty, principal diagnosis, and notably, residence in an old-age home, significantly modify the weekend effect in ways that challenge simple explanations. The moderating effect of old-age home residence directly contradicted our initial expectations, revealing protective effects for emergency readmission outcomes rather than the more pronounced negative impact we had anticipated. For 7-day post-discharge emergency readmission, weekend care transitions were protective only for old-age home residents (aOR 0.962), while showing no significant effect for community-dwellers. Similarly, for 30-day emergency readmission, the protective effect was more pronounced in the old-age home population (aOR 0.969 vs. aOR 0.992). This suggests that the structured environment of old-age homes, with consistent medication management, professional monitoring, and coordinated care, may be particularly effective at mitigating emergency readmission risks when coupled with weekend discharges, possibly due to more seamless care transitions when staff are already present^35^. However, this protective pattern for emergency readmission did not extend equally to mortality outcomes. Regarding 30-day post-discharge mortality, community-dwelling patients had a significant protective association (aOR 0.958), whereas the association was not significant among residents of old-age homes (aOR 0.980). This divergence suggests that while institutional care can effectively prevent disease exacerbations requiring emergency readmission, it provides limited protection against the fundamental biological vulnerability that leads to mortality in this frail population^30,36^.

The significant variations in weekend effects across clinical specialties and diagnostic categories reveal fundamental insights about healthcare system functioning. The protective associations observed in Internal Medicine and Surgery, contrasted with the elevated risks in Emergency Medicine, suggest that the weekend effect is not primarily about temporal variations in care quality, but rather reflects deeper structural factors^5,7^. The consistent protective effects for patients with neoplasms, infectious diseases, and repertory diseases indicate that conditions with established care pathways and dedicated specialist teams can maintain care continuity regardless of discharge timing. Conversely, the increased risk for musculoskeletal conditions highlights how diseases requiring complex rehabilitation planning and multi-disciplinary coordination are particularly vulnerable to weekend care transitions^37^.

These findings collectively suggest that the weekend effect functions not as a simple temporal phenomenon but as a revealing indicator of underlying systemic strengths and vulnerabilities in care coordination. The effect represents the net balance of competing factors: documented limitations in weekend resources, selective patient mortality, clinical decision-making in discharge timing, and the variable robustness of condition-specific support systems. The protective associations observed in certain subgroups likely occur where structural advantages, such as integrated clinical pathways or structured post-acute care environments, outweigh potential weekend-related disadvantages. This understanding necessitates a fundamental reorientation of quality improvement efforts: rather than focusing on avoiding weekend discharges, interventions should prioritize building resilient care transition mechanisms, particularly for conditions requiring multi-specialty management^38^. Additionally, the clinical significance of these findings warrants scrutiny. While our study identified a small protective effect (4.2% relative reduction in 30-day emergency readmission odds), prior work reported similarly narrow effect sizes, including a 4% increased relative risk^11,39^. Though statistically significant, such small effect size in large sample size, exceeding 2.6 million, provides substantial statistical power, thereby enabling the detection of even minor effects as statistically significant^40^, as well as raises questions about their translational clinical relevance^41^.

The extensive territory-wide electronic health dataset covering a decade (2012–2021) provides robust statistical power and generalizability within the context of Hong Kong’s aging population. We identified two distinct hospitalization patterns: weekend-admission/discharge and weekday-admission-discharge. This framework integrates weekend admissions, LOS, and discharges, offering a unique perspective for understanding the weekend effect on mortality and emergency readmission risks, while also highlighting potential non-randomness between these patterns as determined by LOS and the admission-discharge cycle. Several limitations should be acknowledged. First, the reliance on administrative data restricted our ability to capture critical clinical details, such as illness severity scores and functional status. To mitigate this limitation, we utilized LOS strata (7-day module), clinical specialties, and ICD-10 diagnostic codes as proxies for disease severity and systematic differences^16,42^. We also conducted multiple subgroup analyses to assess potential variations in these factors. Second, our dataset is restricted to readmissions and deaths occurring within public hospitals; events in private facilities and out-of-hospital deaths are not captured. Moreover, readmission analyses were limited to emergency readmissions. Elective readmissions were not included, which may partially account for the observed protective post-discharge associations if weekend-admission/discharge disproportionately comprised patients whose subsequent care needs were met through elective rather than emergency pathways. Nevertheless, public hospitals serve as the predominant providers of inpatient care for serious illness in Hong Kong, and the majority of deaths in this population occur in-hospital rather than at home or in alternative care settings^43^. Third, a significant methodological limitation concerns the differential volume of discharges across days of the week. Discharges are substantially less frequent on weekends, particularly Sunday. Because in-hospital deaths are distributed relatively uniformly across days, the weekend-admission/discharge group experiences denominator compression that may partially inflate mortality estimates. Moreover, weekend discharges likely represent a selected cohort enriched for either low-acuity survivors or patients who died in-hospital, a selection pattern that is itself a consequence of reduced weekend staffing and ancillary services. Although LOS stratification and multivariable adjustment partially address this bias, residual confounding by unmeasured illness severity remains a concern. Fourth, the observational design precludes causal inference, leaving residual confounding possible despite multivariable adjustments, including the illness severity despite using the LOS as the proxy^39^ and unmeasured post-discharge factors (e.g., caregiver support, outpatient follow-up adherence^44^, and hospice use^45^ may influence outcomes but were not captured in the dataset. Fifth, the study’s Hong Kong-specific context limits generalizability to other healthcare systems with differing staffing models, resource availability, or end-of-life care practices. Lastly, the small effect sizes for readmission risk reduction, though statistically significant, raise questions about clinical relevance, underscoring the need for cautious interpretation and further investigation into the multifactorial drivers of readmissions.

In summary, this study demonstrates a critical duality in the weekend effect for emergency hospitalizations: weekend admission or discharge is significantly associated with increased in-hospital mortality, yet predicts minimally reduced post-discharge mortality and emergency readmission risk. These paradoxical outcomes are substantially modified by LOS and clinical context, with the protective effects concentrated among patients with longer hospitalizations and those in specific clinical specialties. The findings suggest that the weekend effect primarily reflects a combination of systemic care integration challenges and differential discharge patterns. Future research using prospective designs or causal inference methods is needed to disentangle the contributions of care processes from selection mechanisms. Quality improvement efforts may consider developing resilient care systems with enhanced cross-specialty coordination and condition-specific care pathways that maintain continuity across all transitions, regardless of their timing.

## Supplementary Information

Below is the link to the electronic supplementary material.

Supplementary Material 1

## Data Availability

The datasets for the current study are available from the corresponding author upon reasonable request, contingent upon receiving approval from the Hong Kong Hospital Authority.
