# Supplementary material for: Weekend effect in emergency hospitalizations of older adults in Hong Kong
Source: Sci Rep. 2026 May 26;16:24082. doi: 10.1038/s41598-026-54977-0 (PMC13439168; doi:10.1038/s41598-026-54977-0)
Supplement: Supplementary file 1 [file 41598_2026_54977_MOESM1_ESM.docx]

**Supplementary materials**

**Supplementary Figure 1**. Data selection and analysis processes

**Supplementary Table 1**. The modulo-7 pattern of weekend admission or discharge based on length of stay (1 to 28 days as an example)

| Length of stays, *days* | Stratum | Modulo-7 | Admission day | Corresponding discharge day | Description | Excepted exposure probability | Actual exposure probability |
| --- | --- | --- | --- | --- | --- | --- | --- |
| 1 | 1 | 1 | 5,6,7 | 6,7,1 | Fri admission → Sat discharge, Sat → Sun, Sun → Mon | 3/7, 42.9% | 216803/615929, 35.2% |
| 2 | 1 | 2 | 4,5,6,7 | 6,7,1,2 | Thu → Sat, Fri → Sun, Sat → Mon, Sun → Tue | 4/7, 57.1% | 339585/672927, 50.5% |
| 3 | 1 | 3 | 3,4,6,7 | 6,7,1,2 | Wed → Sat, Thu → Sun, Sat → Tue, Sun → Wed | 4/7, 57.1% | 254216/510003, 49.8% |
| 4 | 1 | 4 | 2,3,6,7 | 6,7,1,2 | Tue → Sat, Wed → Sun, Sat → Wed, Sun → Thu | 4/7, 57.1% | 170047/364521, 46.6% |
| 5 | 1 | 5 | 1,2,6,7 | 6,7,1,2 | Mon → Sat, Tue → Sun, Sat → Thu, Sun → Fri | 4/7, 57.1% | 119220/262260, 45.5% |
| 6 | 1 | 6 | 1,6,7 | 7,1,2 | Mon → Sun, Sat → Fri, Sun → Sat | 3/7, 42.9% | 59668/194030, 30.8% |
| 7 | 1 | 7 | 6,7 | 6,7 | Sat → Sat, Sun → Sun | 2/7, 28.6% | 24635/151904, 16.2% |
| 8 | 2 | 1 | 5,6,7 | 6,7,1 | Fri → Sat, Sat → Sun, Sun → Mon | 3/7, 42.9% | 36685/114889, 31.9% |
| 9 | 2 | 2 | 4,5,6,7 | 6,7,1,2 | Thu → Sat, Fri → Sun, Sat → Mon, Sun → Tue | 4/7, 57.1% | 43465/88448, 49.1% |
| 10 | 2 | 3 | 3,4,6,7 | 6,7,1,2 | Wed → Sat, Thu → Sun, Sat → Tue, Sun → Wed | 4/7, 57.1% | 35848/72067, 49.7% |
| 11 | 2 | 4 | 2,3,6,7 | 6,7,1,2 | Tue → Sat, Wed → Sun, Sat → Wed, Sun → Thu | 4/7, 57.1% | 29070/59198, 49.1% |
| 12 | 2 | 5 | 1,2,6,7 | 6,7,1,2 | Mon → Sat, Tue → Sun, Sat → Thu, Sun → Fri | 4/7, 57.1% | 23825/48612, 49% |
| 13 | 2 | 6 | 1,6,7 | 7,1,2 | Mon → Sun, Sat → Fri, Sun → Sat | 3/7, 42.9% | 13596/41267, 32.9% |
| 14 | 2 | 7 | 6,7 | 6,7 | Sat → Sat, Sun → Sun | 2/7, 28.6% | 6136/35705, 17.2% |
| 15 | 3 | 1 | 5,6,7 | 6,7,1 | Fri → Sat, Sat → Sun, Sun → Mon | 3/7, 42.9% | 9694/30251, 32% |
| 16 | 3 | 2 | 4,5,6,7 | 6,7,1,2 | Thu → Sat, Fri → Sun, Sat → Mon, Sun → Tue | 4/7, 57.1% | 13117/26523, 49.5% |
| 17 | 3 | 3 | 3,4,6,7 | 6,7,1,2 | Wed → Sat, Thu → Sun, Sat → Tue, Sun → Wed | 4/7, 57.1% | 11507/22737, 50.6% |
| 18 | 3 | 4 | 2,3,6,7 | 6,7,1,2 | Tue → Sat, Wed → Sun, Sat → Wed, Sun → Thu | 4/7, 57.1% | 10224/20445, 50% |
| 19 | 3 | 5 | 1,2,6,7 | 6,7,1,2 | Mon → Sat, Tue → Sun, Sat → Thu, Sun → Fri | 4/7, 57.1% | 8975/17869, 50.2% |
| 20 | 3 | 6 | 1,6,7 | 7,1,2 | Mon → Sun, Sat → Fri, Sun → Sat | 3/7, 42.9% | 5576/16004, 34.8% |
| 21 | 3 | 7 | 6,7 | 6,7 | Sat → Sat, Sun → Sun | 2/7, 28.6% | 2605/14397, 18.1% |
| 22 | 4 | 1 | 5,6,7 | 6,7,1 | Fri → Sat, Sat → Sun, Sun → Mon | 3/7, 42.9% | 4090/12693, 32.2% |
| 23 | 4 | 2 | 4,5,6,7 | 6,7,1,2 | Thu → Sat, Fri → Sun, Sat → Mon, Sun → Tue | 4/7, 57.1% | 5726/11622, 49.3% |
| 24 | 4 | 3 | 3,4,6,7 | 6,7,1,2 | Wed → Sat, Thu → Sun, Sat → Tue, Sun → Wed | 4/7, 57.1% | 5367/10600, 50.6% |
| 25 | 4 | 4 | 2,3,6,7 | 6,7,1,2 | Tue → Sat, Wed → Sun, Sat → Wed, Sun → Thu | 4/7, 57.1% | 4573/9262, 49.4% |
| 26 | 4 | 5 | 1,2,6,7 | 6,7,1,2 | Mon → Sat, Tue → Sun, Sat → Thu, Sun → Fri | 4/7, 57.1% | 4310/8307, 51.9% |
| 27 | 4 | 6 | 1,6,7 | 7,1,2 | Mon → Sun, Sat → Fri, Sun → Sat | 3/7, 42.9% | 2829/7929, 35.7% |
| 28 | 4 | 7 | 6,7 | 6,7 | Sat → Sat, Sun → Sun | 2/7, 28.6% | 1375/7478, 18.4% |

**Note**:

Admission and discharge day of week coding: 1=Monday, 2=Tuesday, 3=Wednesday, 4=Thursday, 5=Friday, 6=Saturday, 7=Sunday.

Weekend effect exposure is defined as having either admission or discharge occur on a weekend (Saturday or Sunday).

Expected exposure probabilities were mathematically derived for each individual length of stay (1-28, days) based on weekend admission and discharge patterns, while actual probabilities were calculated as the observed proportion of exposed patients at each specific length of stay in our study.

**Supplementary Table 2**. In-hospital mortality rates by day of admission and discharge, and length of stay

| **Day of week/length of stay** | **Spells** | **In-hospital death** | **Rate (%)** |
| --- | --- | --- | --- |
| **Admission day** |  |  |  |
| Monday | 514276 | 21571 | 4.19 |
| Tuesday | 501392 | 21107 | 4.21 |
| Wednesday | 494304 | 21133 | 4.28 |
| Thursday | 499681 | 21630 | 4.33 |
| Friday | 500582 | 21634 | 4.32 |
| Saturday | 479309 | 21314 | 4.45 |
| Sunday | 458333 | 20705 | 4.52 |
| **Discharge day** |  |  |  |
| Monday | 552976 | 21200 | 3.83 |
| Tuesday | 585888 | 21804 | 3.72 |
| Wednesday | 560174 | 21612 | 3.86 |
| Thursday | 545803 | 21191 | 3.88 |
| Friday | 555313 | 21145 | 3.81 |
| Saturday | 409893 | 21028 | 5.13 |
| Sunday | 237830 | 21114 | 8.88 |
| **Length of stay, *days*** |  |  |  |
| 1 | 615929 | 22098 | 3.59 |
| 2 | 672927 | 14749 | 2.19 |
| 3 | 510003 | 12674 | 2.49 |
| 4 | 364521 | 11041 | 3.03 |
| 5 | 262260 | 9864 | 3.76 |
| 6 | 194030 | 8914 | 4.59 |
| 7 | 151904 | 7586 | 4.99 |
| 8 | 114889 | 6849 | 5.96 |
| 9 | 88448 | 6112 | 6.91 |
| 10 | 72067 | 5419 | 7.52 |
| 11 | 59198 | 4824 | 8.15 |
| 12 | 48612 | 4301 | 8.85 |
| 13 | 41267 | 3906 | 9.47 |
| 14 | 35705 | 3477 | 9.74 |
| 15 | 30251 | 3164 | 10.46 |
| 16 | 26523 | 2991 | 11.28 |
| 17 | 22737 | 2612 | 11.49 |
| 18 | 20445 | 2494 | 12.2 |
| 19 | 17869 | 2242 | 12.55 |
| 20 | 16004 | 2047 | 12.79 |
| 21 | 14397 | 1846 | 12.82 |
| 22 | 12693 | 1746 | 13.76 |
| 23 | 11622 | 1625 | 13.98 |
| 24 | 10600 | 1476 | 13.92 |
| 25 | 9262 | 1383 | 14.93 |
| 26 | 8307 | 1264 | 15.22 |
| 27 | 7929 | 1192 | 15.03 |
| 28 | 7478 | 1198 | 16.02 |

**Supplementary Table 3**. Sensitivity analysis for the association between weekend admissions or discharges and the risk of mortality and readmission

| **Outcomes** | **Propensity score matching** | | **Excluding holiday** | | **Randomly selecting** | |
| --- | --- | --- | --- | --- | --- | --- |
|  | Odds ratio  (95% Confidence interval) | *p*-value | Odds ratio  (95% Confidence interval) | *p*-value | Odds ratio  (95% Confidence interval) | *p*-value |
| In-hospital mortality | 1.371 (1.354-1.387) | <0.001 | 1.368 (1.353-1.384) | <0.001 | 1.352 (1.327-1.377) | <0.001 |
| Morality at 7-day post-discharge | 0.983 (0.948-1.019) | 0.348 | 0.948 (0.916-0.981) | 0.002 | 0.948 (0.902-0.997) | 0.039 |
| Morality at 30-day post-discharge | 0.970 (0.954-0.986) | <0.001 | 0.960 (0.945-0.976) | <0.001 | 0.952 (0.930-0.975) | <0.001 |
| Readmission at 7-day post-discharge | 0.998 (0.987-1.009) | 0.721 | 0.993 (0.983-1.004) | 0.215 | 0.992 (0.973-1.012) | 0.429 |
| Readmission at 30-day post-discharge | 0.985 (0.978-0.992) | <0.001 | 0.987 (0.980-0.994) | <0.001 | 0.972 (0.960-0.985) | <0.001 |

**Note**:

Three sensitivity analyses were conducted. First, propensity score matching was performed using 1:1 nearest-neighbor matching with a caliper of 0.2 to balance covariates between weekday admissions and discharges and weekend admissions or discharges. Second, a sensitivity analysis was conducted excluding public holiday dates. Finally, a sensitivity analysis was performed by randomly selecting one hospitalization spell per patient.

The association between weekend admissions or discharges and the risk of mortality and readmission was evaluated using a logistic regression model that included a random effect for hospitals and adjusted for length of stay, age, sex, old-age home residence, payment source, admission season, clinical specialty, and principal diagnosis.

Readmission risks were assessed after excluding deaths occurring within 7 or 30 days to eliminate competing risks.

**Supplementary Table 4**. Sensitivity analysis for the association between weekend admissions or discharges and the risk of post-discharge mortality and readmission by the stratum of length of day and clinical specialty

| **Subgroup** | **Post-discharge outcomes** | **Propensity score matching** | | **Excluding holiday** | | **Randomly selecting** | |
| --- | --- | --- | --- | --- | --- | --- | --- |
|  |  | Odds ratio  (95% Confidence interval) | *p*-value | Odds ratio  (95% Confidence interval) | *p*-value | Odds ratio  (95% Confidence interval) | *p*-value |
| **Stratum of length of stay** |  |  |  |  |  |  |  |
| Stratum 1 (1–7 days) | Morality at 7-day | 1.043 (0.997-1.090) | 0.065 | 1.024 (0.981-1.069) | 0.282 | 1.028 (0.964-1.096) | 0.398 |
|  | Morality at 30-day | 1.025 (1.005-1.046) | 0.015 | 1.032 (1.012-1.053) | 0.002 | 1.023 (0.993-1.054) | 0.138 |
|  | Readmission at 7-day | 1.020 (1.008-1.033) | 0.001 | 1.019 (1.007-1.031) | 0.002 | 1.018 (0.995-1.041) | 0.120 |
|  | Readmission at 30-day | 1.004 (0.996-1.012) | 0.321 | 1.009 (1.001-1.017) | 0.023 | 1.000 (0.986-1.015) | 0.946 |
| Stratum 2 (8–14 days) | Morality at 7-day | 0.929 (0.863-1.001) | 0.052 | 0.913 (0.851-0.979) | 0.011 | 0.933 (0.842-1.033) | 0.182 |
|  | Morality at 30-day | 0.938 (0.904-0.972) | <0.001 | 0.937 (0.905-0.969) | <0.001 | 0.935 (0.889-0.983) | 0.009 |
|  | Readmission at 7-day | 0.930 (0.903-0.958) | <0.001 | 0.930 (0.904-0.957) | <0.001 | 0.942 (0.895-0.991) | 0.022 |
|  | Readmission at 30-day | 0.938 (0.920-0.957) | <0.001 | 0.943 (0.926-0.961) | <0.001 | 0.927 (0.896-0.959) | <0.001 |
| Stratum 3 (15–21 days) | Morality at 7-day | 0.971 (0.855-1.102) | 0.650 | 0.907 (0.807-1.019) | 0.100 | 0.840 (0.712-0.992) | 0.040 |
|  | Morality at 30-day | 0.914 (0.859-0.972) | 0.004 | 0.903 (0.853-0.956) | <0.001 | 0.850 (0.783-0.924) | <0.001 |
|  | Readmission at 7-day | 0.949 (0.900-1.001) | 0.053 | 0.939 (0.894-0.986) | 0.011 | 0.963 (0.884-1.050) | 0.394 |
|  | Readmission at 30-day | 0.945 (0.911-0.980) | 0.003 | 0.935 (0.904-0.967) | <0.001 | 0.925 (0.871-0.982) | 0.010 |
| Stratum 4 (22–28 days) | Morality at 7-day | 0.945 (0.778-1.146) | 0.564 | 0.886 (0.743-1.056) | 0.177 | 0.898 (0.701-1.150) | 0.393 |
|  | Morality at 30-day | 0.866 (0.790-0.950) | 0.002 | 0.847 (0.778-0.921) | <0.001 | 0.892 (0.791-1.004) | 0.059 |
|  | Readmission at 7-day | 0.943 (0.870-1.021) | 0.148 | 0.920 (0.856-0.990) | 0.025 | 0.939 (0.828-1.066) | 0.331 |
|  | Readmission at 30-day | 0.922 (0.872-0.974) | 0.004 | 0.926 (0.880-0.973) | 0.003 | 0.889 (0.814-0.970) | 0.008 |
| **Clinical specialty** |  |  |  |  |  |  |  |
| Emergency Medicine | Morality at 7-day | 0.937 (0.774-1.135) | 0.507 | 0.882 (0.733-1.063) | 0.187 | 0.696 (0.521-0.930) | 0.014 |
|  | Morality at 30-day | 0.995 (0.912-1.084) | 0.900 | 0.975 (0.897-1.061) | 0.562 | 0.950 (0.838-1.076) | 0.419 |
|  | Readmission at 7-day | 1.042 (1.008-1.076) | 0.014 | 1.048 (1.015-1.083) | 0.004 | 1.088 (1.025-1.154) | 0.006 |
|  | Readmission at 30-day | 1.020 (0.999-1.042) | 0.065 | 1.029 (1.008-1.050) | 0.007 | 1.043 (1.003-1.085) | 0.034 |
| Internal Medicine | Morality at 7-day | 1.002 (0.962-1.043) | 0.942 | 0.971 (0.934-1.010) | 0.140 | 1.002 (0.945-1.062) | 0.954 |
|  | Morality at 30-day | 0.980 (0.961-0.999) | 0.038 | 0.975 (0.957-0.993) | 0.007 | 0.979 (0.952-1.007) | 0.137 |
|  | Readmission at 7-day | 0.993 (0.979-1.007) | 0.331 | 0.988 (0.975-1.001) | 0.073 | 0.968 (0.943-0.993) | 0.013 |
|  | Readmission at 30-day | 0.983 (0.974-0.991) | <0.001 | 0.984 (0.975-0.992) | <0.001 | 0.967 (0.951-0.983) | <0.001 |
| Orthopedics | Morality at 7-day | 0.963 (0.793-1.170) | 0.705 | 0.921 (0.771-1.101) | 0.366 | 0.854 (0.660-1.106) | 0.232 |
|  | Morality at 30-day | 0.954 (0.874-1.041) | 0.286 | 0.942 (0.869-1.022) | 0.150 | 0.958 (0.853-1.075) | 0.462 |
|  | Readmission at 7-day | 0.986 (0.944-1.029) | 0.511 | 0.994 (0.955-1.035) | 0.777 | 1.087 (1.013-1.167) | 0.021 |
|  | Readmission at 30-day | 0.996 (0.969-1.024) | 0.784 | 0.992 (0.967-1.018) | 0.562 | 0.972 (0.928-1.018) | 0.233 |
| Surgery | Morality at 7-day | 0.955 (0.856-1.066) | 0.411 | 0.889 (0.801-0.987) | 0.027 | 0.854 (0.741-0.985) | 0.030 |
|  | Morality at 30-day | 0.972 (0.926-1.020) | 0.247 | 0.953 (0.909-0.998) | 0.041 | 0.927 (0.869-0.988) | 0.021 |
|  | Readmission at 7-day | 1.007 (0.981-1.034) | 0.603 | 0.995 (0.970-1.021) | 0.728 | 0.988 (0.945-1.034) | 0.612 |
|  | Readmission at 30-day | 0.980 (0.962-0.998) | 0.030 | 0.980 (0.963-0.997) | 0.023 | 0.961 (0.932-0.991) | 0.011 |
| Others | Morality at 7-day | 0.872 (0.742-1.024) | 0.094 | 0.882 (0.757-1.027) | 0.106 | 0.793 (0.644-0.976) | 0.028 |
|  | Morality at 30-day | 0.849 (0.786-0.917) | <0.001 | 0.847 (0.787-0.912) | <0.001 | 0.758 (0.685-0.838) | <0.001 |
|  | Readmission at 7-day | 0.924 (0.865-0.988) | 0.021 | 0.904 (0.849-0.964) | 0.002 | 0.957 (0.866-1.057) | 0.386 |
|  | Readmission at 30-day | 0.903 (0.862-0.946) | <0.001 | 0.907 (0.868-0.949) | <0.001 | 0.895 (0.834-0.960) | 0.002 |

**Note**:

Three sensitivity analyses were conducted. First, propensity score matching was performed using 1:1 nearest-neighbor matching with a caliper of 0.2 to balance covariates between weekday admissions and discharges and weekend admissions or discharges. Second, a sensitivity analysis was conducted excluding public holiday dates. Finally, a sensitivity analysis was performed by randomly selecting one hospitalization spell per patient.

The association between weekend admissions or discharges and the risk of mortality and readmission was evaluated using a logistic regression model that included a random effect for hospitals and adjusted for length of stay, age, sex, old-age home residence, payment source, admission season, clinical specialty, and principal diagnosis.

Readmission risks were assessed after excluding deaths occurring within 7 or 30 days to eliminate competing risks.
